# Genetic influences on the onset of obstructive sleep apnoea and daytime sleepiness: a twin study

**DOI:** 10.1186/s12931-019-1095-x

**Published:** 2019-06-17

**Authors:** Marcell Szily, Adam D. Tarnoki, David L. Tarnoki, Daniel T. Kovacs, Bianka Forgo, Jooyeon Lee, Eunae Kim, Joohon Sung, Laszlo Kunos, Martina Meszaros, Veronika Muller, Andras Bikov

**Affiliations:** 1 Department of Radiology, Semmelweis University, 78/A Ulloi street, 1082 Budapest, Hungary; 2 Complex Disease and Genome Epidemiology Branch, Department of Public Health Science, School of Public Health, Seoul National University, Seoul, Republic of Korea; 3 Department of Pulmonology, Semmelweis University, Budapest, Hungary

**Keywords:** Sleep apnoea, Sleepiness, Environment, Heritability

## Abstract

**Background:**

Obstructive sleep apnoea (OSA) is one of the major sources of the excessive daily sleepiness, cognitive dysfunction, and it increases cardiovascular morbidity and mortality. Previous studies suggested a possible genetic influence, based on questionnaires but no objective genetic study was conducted to understand the exact variance underpinned by genetic factors.

**Methods:**

Seventy-one Hungarian twin pairs involved from the Hungarian Twin Registry (48 monozygotic, MZ and 23 dizygotic, DZ pairs, mean age 51 ± 15 years) underwent overnight polysomnography (Somnoscreen Plus Tele PSG, Somnomedics GMBH, Germany). Apnoea hypopnea index (AHI), respiratory disturbance index (RDI) and oxygen desaturation index (ODI) were registered. Daytime sleepiness was measured with the Epworth Sleepiness Scale (ESS). Bivariate heritability analysis was applied.

**Results:**

The prevalence of OSA was 41% in our study population. The heritability of the AHI, ODI and RDI ranged between 69% and 83%, while the OSA, defined by an AHI ≥5/h, was itself 73% heritable. The unshared environmental component explained the rest of the variance between 17% and 31%. Daytime sleepiness was mostly determined by the environment, and the variance was influenced in 34% by the additive genetic factors. These associations were present after additional adjustment for body mass index.

**Conclusion:**

OSA and the indices of OSA severity are heritable, while daytime sleepiness is mostly influenced by environmental factors. Further studies should elucidate whether close relatives of patients with OSA may benefit from early family risk based screening.

## Introduction

Obstructive Sleep Apnoea (OSA) is a common disorder which is associated with day and night time symptoms, cognitive deficit and increased risk for cardiovascular and metabolic morbidity and mortality. OSA is characterized by partial or total collapse of the upper airways leading to intermittent oxygen desaturation and frequent awakenings. The anatomical abnormalities and variabilities are an important part of the pathogenesis of the obstructive sleep apnoea. MRI studies showed that the structure of upper airway system, especially maxillary, mandibular size and body length can increase the vulnerability to OSA [1]. Furthermore, the accumulation of fatty tissue in the parapharyngeal area and increased body weight decreases the diameter of the upper airway system, which is related to the collapse [2]. It is known that besides the craniofacial anatomy, impaired neural control and upper airway muscle myopathy can also increase the risk for OSA [3]. According to the current concept, both genetic susceptibility and environmental factors contribute to disease development, however, the extent of genetic predisposition is still unclear.

Patel et al. conducted a family study which proved that the smallest surface diameter of the oropharynx is a heritable trait [4]. The same working group described the heritability of OSA in another family study [5]. Compared to family studies, twin studies can better discriminate heritable and acquired factors, as family members share common environments which makes them unable to disentangle potential genetic and environmental influences. On the other hand, twin studies can take the shared family environment into account, including potentially deleterious and confounding effects (eg., salt intake, alcohol use, and lack of physical exercise). Only two twin studies have been performed in this field, one assessed the role of hereditary factors in the background of snoring [6], the other one the inheritance of OSA, estimating it to be up to 50% [7], however, the results were based on a questionnaire survey and OSA was not confirmed and its severity has not been evaluated by a diagnostic sleep study.

In our study, we measured the sleep parameters by polysomnography in a sample of adult twins in order to understand how much genetic predisposition exists in the pathogenesis of the various parameters of obstructive sleep apnoea.

## Materials and methods

Seventy-one Hungarian twin pairs (48 monozygotic, MZ and 23 dizygotic, DZ) were recruited from the Hungarian Twin Registry [8]. None of the participants were previously diagnosed with OSA and have not received any treatment, including positive airway pressure therapy or mandibular advancement device. Pregnant subjects, patients with uncontrolled chronic cardiorespiratory disease (i.e. asthma exacerbation or acute heart failure) and those with an acute respiratory infection within 4 weeks of measurement were excluded. The population consisted participants of Caucasian ethnicity. Zygosity was assessed using a standard questionnaire [9]. Smoking history was recorded: each subject was categorized as never, former or active smoker. Pack-years were calculated as Number of pack years = (number of cigarettes smoked per day × number of years smoked)/20. Weight was measured by OMRON BF500 monitor (Omron Healthcare Ltd., Kyoto, Japan). Body mass index (BMI) was determined by the weight (kg)/height (m)^2^.

Subjects underwent an overnight polysomnography (PSG) at the Department of Pulmonology, Semmelweis University. Before the PSG, twin pairs completed the Epworth Sleepiness Scale (ESS) questionnaire, medical history was taken, blood pressure and heart rate were recorded. PSG was performed using the Somnoscreen Plus Tele PSG (Somnomedics GmbH, Germany) according to the international recommendation [10]. Accordingly, electroencephalography, electrooculography and electromyography, body position, chest and abdominal movements, intranasal pressure, electrocardiography and oxygen saturation were recorded. Sleep stages and arousals, movements and cardiopulmonary events were manually scored based on the recommendations of the American Academy of Sleep Medicine (AASM) [11]. Total sleep time (TST), percentage of sleep time spent with oxygen saturation below90% (TST90%), minimal oxygen saturation (MinSatO_2_), arousal index (AI), Respiratory arousal index (RespAI) were registered. Apnea-hypopnea index (AHI), the respiratory disturbance index (RDI) and the oxygen desaturation index (ODI) were calculated to determine the presence and severity of OSA. In the morning following the study, blood pressure and heart rate were recorded within 1 hour after the rising.

The research was conducted in accordance with the Declaration of Helsinki. The local ethical committee (Semmelweis University TUKEB 30/2014) approved the study and all subjects gave informed consent prior to study entry.

### Statistical analysis

Descriptive statistics (mean ± standard deviation for continuous variables, percentage for categorical variables) were computed. Linear and logistic regression analysis was used to identify variables independently associated with AHI, RDI and ODI. AHI, RDI and ODI were log transformed due to not normal distribution.

Structural equation modelling was performed to estimate the variance components of the ACE model which partitions variance is due to additive genetic effects (A or h2: heritability estimates), common environmental effects (C or c2: proportion of variance explained by common environments) and other residual effects (E or e2, including SE: standard error). The A measures the effects due to genes at multiple loci or multiple alleles at one locus. The C estimates the contribution of the common family environment of both twins (familiar socialization, diet, exposure to high levels of air pollution, shared womb, etc.), whereas the unshared environmental component estimates the effects that apply only to each individual twin and includes measurement error. If A was 0, CE model was applied. Heritability analyses were conducted using the variance component models implemented in SOLAR Eclipse version 8.1.1 by the members of the Korean Twin Registry, which analyses the differences of variances according to family structure [12]. Univariate heritability was estimated to assess heritability of each of the single traits. Heritability was estimated using proportion that is explained by additive genetic effects over the total phenotypic variance, after adjusting for potential confounding factors such as age and sex as fixed effects. Post-hoc adjustment for BMI has also been performed. The C was modelled based on their family IDs.

## Results

### Clinical characteristics and measures

As shown in Tables 1, 68% of all twins were monozygotic and 32% were dizygotic, with females representing 73% of the study sample. The subjects had in average normal BMI (mean BMI: 24.1 kg/m^2^). OSA was diagnosed in 58 subjects (41%), of whom 44 had mild (AHI 5–15/h), 12 had moderate (AHI 15–30/h) and 2 had severe (AHI > 30/h) disease. Patients with moderate-to-severe disease were offered with a continuous positive airway pressure (CPAP) therapy, however their progress has not been evaluated as part of the study.

**Table 1:** Characterisitcs of the study population (*n* = 142)

Characteristics	Mean (SD)/N(%)
Number of twins	
MZ twins	48 pairs (67.6%)
DZ twins	23 pairs (32.4%)
Sex	
Male	40 (28.6%)
Female	102 (72.9%)
Age [years]	50.5 (15.5)
Body mass index (BMI) [kg/m^2^]	24.1 (3.6)
Diabetes mellitus (DM)	16 (11.30%)
Hypertension	60 (42.3%)
Hypercholesterinaemia	55 (39.3%)
Epworth sleepiness scale (ESS)	6.2 (3.6)
Apnoe hypopnoe index (AHI)	6.4 (7.0)
Arousal index (AI)	48.0 (21.3)
Respiratory arousal index	1.33 (1.77)
Minimal oxygen saturation (MinSatO_2_)	88.5 (4.6)
Oxygen desaturation index (ODI)	5.0 (6.8)
Respiratory disturbance index (RDI)	14.7 (8.7)
Total Sleep Time (TST)	0.27 (0.03)
TST90	2.3 (6.1)
Presence of obstructive sleep apnea (OSA) based on AHI > 5/h	58 (41.4%)

TST90%: percentage of sleep time spent with oxygen saturation below 90%

Tables 2 and 3 show the regression analysis with risk factors associated with obstructive respiratory indices (logAHI, logRDI, logODI). There was a significant relationship between BMI and RDI as well as ODI (both *p* < 0.01) with a trend for a significant association between BMI and AHI (*p* = 0.07). Age was directly related to all of the indices (all *p* < 0.01). Only higher ODI was associated significantly with male gender, while AHI or RDI did not correlate with gender. There was no relationship between smoking history and obstructive respiratory events (all *p* > 0.05).

**Table 2:** Association of parameters related to sleep disturbance with metric risk factors

	logAHI		logRDI		logODI	
	R^2^	P	R^2^	P	R^2^	P
age	0.096	< 0.0001	0.070	0.002	0.290	< 0.0001
BMI	0.086	0.074	0.210	0.003	0.277	0.001

In this table you can observe determination coefficient of regression, R2 and related *p* value

*AHI* apnoea-hypopnoea index, *ODI* oxygen desaturation index, *RDI* respiratory disturbance index, *BMI* body mass index

**Table 3:** Association of parameters related to sleep disturbance to categorical risk factors

	logAHI		logRDI		logODI	
	Wilks’-lambda	P	Wilks’-lambda	P	Wilks’-lambda	P
Sex	0.984	0.206	0.984	0.162	0.963	0.032
Smoking	0.988	0.216	0.979	0.107	0.997	0.539

The calculated values are Wilks’-lambda and associated *p*-value

*AHI* apnoea-hypopnoea index, *ODI* oxygen desaturation index, *RDI* respiratory disturbance index

### Univariate genetic modelling

Comparison of h2 with c2 proved genetic influence on all variables, except for TST, suggesting that genetic factors may be important contributors to these variables (Table 4). We fitted ACE models, in which variance of the observed total phenotypes was partitioned into additive genetic, common environmental and other random effects. Heritability was estimated using proportion that is explained by additive genetic effects over the total phenotypic variance, after adjusting for potential confounding factors such as age and sex as fixed effects. Univariate heritability was estimated to assess the heritability of each of the single traits, and bivariate heritability estimations were conducted to assess genetic and environmental correlations between two variables of interests.

**Table 4:** Heritability estimates of breathing-related sleep disorder indices in a case of Model1 and Model2

	h^2^ (SE)	c^2^ (SE)	e^2^ (SE)
AHI	0.73 (0.08)***	0	0.27
AI	0.55 (0.13)***	0	0.45
ESS	0.34 (0.38)*	0.31 (0.36)	0.66
MinSatO_2_	0.96 (0.01)***	0	0.04
ODI	0.83 (0.05)***	0	0.17
OSA	0.73 (0.15)***	0	0.27
RDI	0.69 (0.09)***	0	0.31
RespAI	0.75 (0.16)*	0	0.25
TST	0	0.25 (0.14)*	0.75
TST90	0.97 (0.008)***	0	0.03

*p < 0.2, ***p < 0.001

Model 1 for age and sex, Model 2 for age, sex and BMI are adjusted

h^2^: heritability estimates; c^2^: proportion of variance explained by common environments; SE: standard error, e^2^: proportion of variance explained by unique environments

Model-2 results did not change after additional adjustment for BMI besides age and sex

For most parameters, the best fitting model was one including the A (h2) and E (e2) components, except Epworth and TST, where ACE or CE model had better fitting. The heritability of the AHI, ODI and RDI ranged between 69 and 83%, while the OSA was itself 73% heritable. The unshared environmental component explained the rest of the variance between 17 and 31%. Shared environmental effects for these measures were not detected. The Epworth sleepiness scale was mostly determined by the environment, and the variance was influenced in 34% by the additive genetic factors. Additional adjustment for BMI besides sex and age in Model-2 had no influence on the results.

## Discussion

This is the first study investigating the heritability of OSA using objective sleep assessment. We reported that the sleep parameters and OSA show a high heritability and unshared environmental factors explain rest of the variance. Adjustment for BMI, age or gender implied that the influence of heritability is independent of any potential influence of these variables.

By comparing the twin correlations, we could estimate the variance explained by additive genetic, shared environmental (e.g., shared womb) and non-shared environmental effects (e.g., lifestyle factors, measurement error). Our results can partially be explained by the inheritance of the smallest surface diameter of the oropharynx, one of the important determinants of OSA, which has been demonstrated in a family study (30–40%), albeit the rate of heritability was less than that we have reported [4]. As factors other than oropharynx diameter (obesity, other anatomical situations such as tongue volume) also play a role in the development of OSA, the heritability of these factors may increase the heredity level in addition to the aforementioned anatomical situation. In another family study, the heredity of the AHI was 34–37%, but our twin study found a much higher inheritance [5]. The reason for this is to be found also in the different test methods, because family studies are suitable for determining the similarity or difference between generations and do not take into account external factors such as family environment and culture, which in turn are taken into account in the twin studies by separating genetic and common environmental factors [13]. The only previous twin study of OSA has been proven to have inheritance around 50%. However, OSA diagnosis was based on a questionnaire survey, which might increase the measurement error (E variance) [7]. Therefore, our present study is the first to investigate the inheritance of OSA with objective overnight PSG and first to show high inheritance.

High inheritance draws attention to the likelihood that the disease might occur in close relatives and descendants of patients with sleep disorders. For this reason, early screening and predisposing, controllable factors such as obesity (e.g. thick neck circumference), smoking, high blood pressure and diabetes are essential to prevent or treat. Non-influenced factors include male gender, ethnicity and age. Treatment is further complicated by hereditary predisposition to body composition, incidence of smoking and blood pressure, in 79 and 51%, according to the previous twin studies [14–16]. This means that the prevention of these predisposing factors is also important, since in genetically predisposed individuals it can be associated with the formation of OSA. Most of these factors are influenced, albeit the genetic predisposition, by epigenetic factors, playing an important role in transcriptional and post-transcriptional regulation, including DNA methylation, histone acetylation, miRNA and transcriptome profiling, non-coding RNA regulation and RNA editing [17, 18]. Due to the high public health importance of OSA, close relatives of patients with OSA should be screened to prevent OSA-related emergence of comorbidities and mortality [19]. Recent publications demonstrated the utility of OSA screening in type 2 diabetes and obesity [20, 21]. Our study draws attention to the fact that screening programs may include family history.

It is evident that obesity, which has a high heritability itself ​​ [14, 16], predisposes to OSA, and the prevalence of OSA is increasing worldwide because of the ongoing epidemic of obesity [22]. Obesity associated OSA has been independently associated with the surrogate markers of cardiovascular risk, including sympathetic activation, systemic inflammation, and endothelial dysfunction [22], however, it was unclear whether this association is genetically linked or not. Our findings show an evidence that BMI is associated with OSA though a non-genetic link which might have a clinical implication that physical activity or any other measure of weight reduction can be more effective in OSA management. A recent study has shown that combined occurrence of obesity and OSA may interact to reduce exercise capacity which highlight the importance of obesity control programs among women [23]. This finding is supported by a recent study demonstrating that OSA patients are less physically active than individuals without OSA [24]. The high heritability of sleep parameters highlights the role of earlier identification of OSA in genetically predisposed individuals, since treatment strategies to improve sleep may contribute to overall health outcomes for patients with obesity [25]. The non-genetic link can be the reason why recent studies showed that effective treatment of OSA with CPAP significantly reduces visceral fat [22, 26].

The average daytime sleepiness reported by the study participants was not clinically significant and even in patients with moderate to severe OSA sleepiness was not alarming to make OSA diagnosis available prior to our study. This is in line with a very recent German population-based study that the proportion of sleepy (ESS ≥ 10) patients within the OSA group is relatively low (15%) [27]. In line with this, unlike OSA, sleepiness was determined by environmental factors. We did not intend to evaluate these factors, but they may include poor sleep hygiene, work shifts, diet and medications.

Potential strengths and limitations of our study should be considered. The main strength is that all PSG measurements were performed by the same personnel and with the same device at one research site. Limitations include relatively small sample size and especially low number of DZ pairs, which prohibited the more precise evaluation of each sleep associated variables (eg. TST). Of note, the study population consisted of patients with relatively mild disease severity. Further twin studies, involving patients with morbid obesity or more severe OSA are warranted to see if this high genetic association is present in these patients. Finally, the heritability model based on twins has been criticized because the gene-environment interplay is very difficult to assess [28]. However, large population twin studies can take into account gene-environment and gene–gene interactions in order to study complex phenotypes.

## Conclusions

In summary, the present study showed for the first time objectively the high genetic effects on obstructive sleep apnoea variables in adult healthy twins, independently of age, gender and body mass index. The relatively low unshared environmental influence still highlights the role for the prevention of known environmental risk factors, particularly through epigenetic effects. These observations provide important insights into the pathogenesis and potential treatment of OSA and stimulate further epigenetic studies to understand interconnections between pathophysiology of sleep and metabolic diseases, including obesity.

## Abbreviations

A: Additive genetic effects
AASM: American Academy of Sleep Medicine
ACE: Classical twin statistical model, A: additive genetic effects, C: common environmental effects, E: unique environmental effects
AHI: Apnoe Hypopnoe Index
AI: Arousal Index
BMI: Body Mass Index
C: Common environmental effects
c2: Proportion of variance explained by common environments
CE: Statistical model in twin studies when A is zero.
CPAP: Continuous positive airway pressure
DM: Diabetes mellitus
DNA: Deoxyribonucleic acid
DZ: Dizygotic
E: Environmental and other random effects
ESS: Epworth Sleepiness Scale
h2: Heritability estimates
MinSatO2: Minimal oxygen saturation
miRNA: Micro ribonucleic acid
MZ: Monozygotic
ODI: Oxygen Desaturation Index
OSA: Obstructive Sleep Apnoe
PSG: Polysomnography
RDI: Respiratory Disturbance Index
rDZ: Correlation in dizygotic twins
RespAI: Respiratory Arousal Index
rMZ: Correlation in monozygotic twins
RNA: Ribonucleic acid
SE: Standard error
TST: Total Sleep Time
TST90%: Percentage of sleep time spent with oxygen saturation below 90%
